# Speech based natural language profile before, during and after the onset of psychosis: A cluster analysis

**DOI:** 10.1111/acps.13685

**Published:** 2024-04-10

**Authors:** Tyler C. Dalal, Liangbing Liang, Angelica M. Silva, Michael Mackinley, Alban Voppel, Lena Palaniyappan

**Affiliations:** ^1^ Schulich School of Medicine and Dentistry Western University London Ontario Canada; ^2^ Robarts Research Institute London Ontario Canada; ^3^ Douglas Mental Health University Institute McGill University Montreal Quebec Canada; ^4^ Department of Psychiatry Western University London Ontario Canada

**Keywords:** communication, computational, disorganization, early‐intervention, impoverishment, linguistics, thought

## Abstract

**Background and Hypothesis:**

Speech markers are digitally acquired, computationally derived, quantifiable set of measures that reflect the state of neurocognitive processes relevant for social functioning. “Oddities” in language and communication have historically been seen as a core feature of schizophrenia. The application of natural language processing (NLP) to speech samples can elucidate even the most subtle deviations in language. We aim to determine if NLP based profiles that are distinctive of schizophrenia can be observed across the various clinical phases of psychosis.

**Design:**

Our sample consisted of 147 participants and included 39 healthy controls (HC), 72 with first‐episode psychosis (FEP), 18 in a clinical high‐risk state (CHR), 18 with schizophrenia (SZ). A structured task elicited 3 minutes of speech, which was then transformed into quantitative measures on 12 linguistic variables (lexical, syntactic, and semantic). Cluster analysis that leveraged healthy variations was then applied to determine language‐based subgroups.

**Results:**

We observed a three‐cluster solution. The largest cluster included most HC and the majority of patients, indicating a ‘typical linguistic profile (TLP)’. One of the atypical clusters had notably high semantic similarity in word choices with less perceptual words, lower cohesion and analytical structure; this cluster was almost entirely composed of patients in early stages of psychosis (EPP – early phase profile). The second atypical cluster had more patients with established schizophrenia (SPP – stable phase profile), with more perceptual but less cognitive/emotional word classes, simpler syntactic structure, and a lack of sufficient reference to prior information (reduced givenness).

**Conclusion:**

The patterns of speech deviations in early and established stages of schizophrenia are distinguishable from each other and detectable when lexical, semantic and syntactic aspects are assessed in the pursuit of ‘formal thought disorder’.

**Significant outcomes**

- There is a systematic variation in linguistic profiles among patients with psychosis; this results in an identifiable number of subgroups with distinct linguistic profiles.
- Most patients (53.7%) clustered along with healthy individuals forming a ‘typical language subgroup’ (Cluster 3). 46.3% (33%–51%) of patients across all stages of illness had an atypical language profile (Clusters 1 and 2).
- One of the two atypical subgroups had no individuals with established schizophrenia (Cluster 2), but a profile exclusive to early stages of psychosis (subthreshold (high‐risk) or suprathreshold (first episode)), with higher load of clinically detectable disorganization and impoverishment among patients with first episode psychosis.

**Limitations**

- Cross‐sectional design limits investigating the stability of reported subgroups.
- While our sample size was sufficiently powered to detect a cluster structure and was larger than most other clustering studies employing a similar variable set, we had disproportionate number of people with first episode psychosis compared to those with clinical high‐risk or established schizophrenia.

## 1 INTRODUCTION

Historically, the presence of a notable ‘oddity’ in everyday language use has been seen as a core feature of schizophrenia.^1^, ^2^, ^3^, ^4^ It is evident that many aspects of language pertinent to referential,^5^, ^6^ propositional^7^ and contextual meaning^8^, ^9^ are affected in schizophrenia. These are reflected in lexical choices that patients make during a discourse,^10^, ^11^ the structure of utterances,^12^, ^13^, ^14^ and cohesion among various meaningful units in a discourse.^4^, ^15^, ^16^ But patients demonstrate varying degrees of these suspected abnormalities in most cross‐sectional studies.^17^, ^18^, ^19^, ^20^ Factors such as the stage of illness (prodromal, first episode and chronic), the presence of affective symptoms^21^, ^22^ such as mania and depression,^23^ the demographic background,^24^, ^25^ and the level of social functioning^26^ appears to affect the degree of reported anomalies. Thus, there may not be a singular characteristic pattern of speech that typifies schizophrenia. In contrast, several distinct patterns of speech may characterize psychosis. Subgroups of patients with similar quantitative linguistic characteristics may share common causal mechanisms for the ‘oddities’ observed. The current work focuses on exploiting this variation to understand language use at the different stages of psychosis through digital acquisition and computerized analysis of speech.

The use of computers to study thought disorders in psychosis has a long history. The use of General Inquirer, a content analysis program, by Maher (for written texts from patients)^27^ and Tucker and Rosenberg on speech recordings pioneered this approach.^28^ Morice and Ingram manually generated syntax trees from speech but subjected them to computerized analysis (PSYCHLAN, on Apple II),^29^, ^30^, ^31^, ^32^ before other linguistic analysis programs were introduced to study speech samples from patients in English^33^ and French.^34^ Despite their nuance, these text mining approaches had limited sensitivity and scalability, without substantial impact on how thought disorder is assessed in the clinic. In part, this was due to the earlier focus on counts and quantities, rather than developing more complete psycholinguistic profiles based on natural language.

Natural language processing (NLP) techniques^35^, ^36^ applied to digitally acquired speech have reduced the dependence on manual rating of speech and language, improving our sensitivity to detect even subtle differences in language behaviors that are hard to recognize by trained clinicians.^36^, ^37^, ^38^, ^39^ In fact, several key NLP markers vary with the severity of clinical symptoms.^35^, ^40^, ^41^ Cluster analysis is a statistical method used to classify individuals with similar behaviors into subgroups. In the context of schizophrenia, clustering based on linguistic variables has resulted in reports of two patient subgroups with lexical or interaction impairment,^42^ fragmented speakers and prolonged pausers,^43^ patients with lower fluency or lower lexical diversity^44^ or 4 subgroups with varying speech complexity. These findings suggest that there are likely linguistic subgroups within schizophrenia.

The above clustering studies in psychosis have two main limitations. First, they exclusively focused on clinically stable, medicated patients, and considered a limited aspect of language such as syntax, acoustics, or pragmatics alone (except Bambini et al.^44^). Consequently, we do not know if different linguistic profiles occur in the pre‐psychotic risk phase (clinically high risk, but subthreshold in terms of symptom severity) and first episode stages (significant symptoms with notable functional impact) of illness. Second, previous case–control studies on NLP variables have shown that there is no clear separation between patients and healthy controls for the variables studied so far. However, most clustering studies (except Schneider et al.^45^) only include patients, assuming a natural distinction between patients and healthy controls. This overlooks the variations among healthy speakers as a potential source of variation among patient subgroups.^46^

We employ NLP variables reflecting the generation of conversational meaning when describing a picture (referent) at various levels: lexical choices, syntactical structure and cohesion in discourse (pragmatics). We investigate the resulting linguistic profiles among individuals with no psychosis (HC), with subthreshold psychosis (clinical high‐risk or CHR), first episode, untreated psychosis (FEP) and established, medicated schizophrenia (SZ). This work sets out to address 3 questions:

1. Is there a systematic variation in linguistic profiles in psychosis? Based on prior clustering work discussed above, we predict that such variation exists and will result in a finite number of subgroups in our sample.
2. Is this variation distinct from the inter‐individual differences seen among non‐psychotic, apparently healthy individuals? We argue that the features of speech among patients that are ascribed to the pathological groups are indeed extremes of healthy distributions that tend to cluster together (see Cohen et al.^47^; Tang et al.^48^, ^49^). We expect many patients to cluster along with HCs, while no subgroups being exclusively comprised of patients alone.
3. Is there a distinct linguistic profile that typifies later versus early stages of psychosis? Based on the known course of clinically rated thought disorder symptoms,^50^, ^51^, ^52^, ^53^ and the recent NLP studies identifying higher semantic/syntactic deviations in early phase of psychosis,^38^ we expect to see a distinct linguistic profile defining early‐stage illness (with some distinction between CHR and FEP who vary in symptom severity).

## 2 MATERIALS AND METHODS

### 2.1 Data collection and clinical assessment

A total of 147 subjects (39 HC, 72 FEP, 18 CHR, 18 SZ) from an ongoing study (Tracking Outcome in Psychosis (TOPSY; ClinicalTrials.gov Identifier: NCT02882204) recruited between 2017 and 2020 were included in this study. Inclusion criteria for TOPSY clinical groups were: age between 16 and 45 years and enrollment in the Prevention and Early Intervention Program for Psychosis. Exclusion criteria included a history of drug or alcohol dependence in the past year, history of head injury, intellectual disability or suffering from medical conditions (such as untreated hypertension, diabetes, hepatic/renal insufficiency, neurological illnesses), or otherwise being unable to provide informed consent.

Patients with first‐episode psychosis (FEP) were recruited from the Prevention and Early Intervention for Psychosis Program (PEPP) at London Health Sciences Centre in London, Ontario, Canada, at the point of first referral to the service. PEPP is a catchment area based, high‐fidelity Early Intervention Program where all patients with first‐episode psychosis in the city of London, ON get referred to; 40% of patients during 2017–2019 were referred during an acute hospital stay. For this study, FEP subjects were assessed within the first week of referral to the first‐episode psychosis team, with a requirement of <2 weeks of lifetime antipsychotic exposure. As such, antipsychotic exposure was <3 days for the defined daily dose (DDD) in the final sample of FEP (calculated by converting various prescribed antipsychotic medication doses to a common equivalent and multiplying by the days of exposure; https://www.whocc.no/atc_ddd_index_and_guidelines/guidelines/).

The CHR group was recruited from the PROSPECT (Prodromal Symptoms of Psychosis – Early Clinical Identification and Treatment, part of the PEPP) program at London Health Sciences Center, London, Ontario. These help‐seeking subjects were evaluated with the Structured Interview for Psychosis‐risk Syndromes (SIPS)^54^ to determine if they met criteria for Attenuated Psychosis Syndrome or brief and limited intermittent psychosis (BLIPS) and were included if they had no prior lifetime antipsychotic exposure.

The SZ group were clinically stable on long‐acting injectable medications with >3 years since illness onset, with no recorded hospitalizations in the past year, and were receiving community‐based care from psychiatrists affiliated with the first‐episode program (PEPP, London, Ontario). Importantly, all subjects were recruited regardless of a prior history of disorganization/thought disorder, thus avoiding a selection bias of the sample towards language‐related symptomatology.

All clinical interviews were conducted by psychiatrists to determine diagnosis and illness severity. Following this, speech samples were obtained through a semi‐standardized interview utilizing the Thought and Language Index by research assistants. The Research Ethics Board at Western University approved all study procedures and subjects were provided with informed consent prior to participating.

### 2.2 Clinical scales

The seven clinical scales included in analysis were the condensed 8‐item positive and negative syndrome scale^55^, ^56^ (PANSS), young mania rating scale^57^ (YMRS), thought and language index^58^ (TLI), brief negative symptom scale^59^ (BNSS), social and occupational functioning assessment score^60^ (SOFAS), calgary depression scale for schizophrenia^61^ (CDS), and the clinical global impressions scale^62^ (CGIS). Additionally, four subscales from the PANSS^63^ (positive symptoms, negative symptoms, disorganization, general symptoms) and two subscales from TLI (impoverishment and disorganization) were included in analysis. All clinical subjects (FEP, CHR, SCZ) were assessed with the seven clinical scales. Thought and Language Index assessment was applied to speech samples from all subjects.

### 2.3 Speech elicitation and processing

Speech and language data was collected by an active (i.e., patient‐controlled, thus not passive listening), prompt‐induced (not spontaneous) speech data acquisition approach using a digital recorder. Both patient and interviewer speech were collected (English dialogues) but only patient data was processed through automated computational linguistic approaches. We used the short version of thought and language index (TLI),^58^ which is an interview‐based instrument providing a reliable assessment of formal thought disorder, able to detect even mild aberrations in speech. A black and white picture prompt was used to enable subjects to speak for 1‐min on the characteristics of a photograph (three photographs total for a total of 3 min) from the thematic apperception test^64^ after hearing the interviewer's instructions to “describe the picture to them, as fully as they can”. The interviewers read a standardized instruction and provided minimal prompting to the individual during their response to ensure validity. Responses were recorded on a digital recorder and transcribed to text. Non‐speech data (i.e., laughing, fillers such as uhm) were excluded, leaving only the participant's speech. Transcriptions were transformed into all‐lowercase sentences, with sentence boundaries determined using manual judgment. We did not include the interviewer's speech as these were infrequent (most subjects did not need them) and minimal when used (e.g., “anything else?”).

### 2.4 Variable selection

Defining a set of variables for clustering analysis is a critical step that can influence the observed outcomes. The variable space is extremely large and prior observations and theoretical guidance is critical for selection. We focused on three levels of meaning‐making devices communicated in everyday language and made our choices based on our prior work.

1. Choice of words or lexical variables: Based on prior works^65^ we focused on the 'analytical thinking index' (reflecting the proportional use of articles, and prepositions in speech), and extending Bambini et al.^44^ the psychological lexicon (including linguistic function words such as pronouns, cognitive, affective, perceptual, and temporal processes). The lexical variables were extracted with the linguistic inquiry and word count^66^ (LIWC‐22). LIWC‐22 provides normalized frequencies of words at conceptual level of information (e.g., “cried” is categorized as a negative emotion word, a verb, and has a focus on the past).
2. Structure of utterances or syntax variables: Based on prior works, we chose syntactic complexity^13^, ^67^ derived from the sum of 5 subscales (see Supplemental Materials) and clause complexity. The syntactic variables were extracted with the tool for automatic analysis of syntactic sophistication and complexity^68^, ^69^ (TAASSC 1.3.8). TAASSC uses reference corpora^70^ to analyze indices related to syntax.
3. Semantic cohesion: Semantic similarity, sometimes called coherence in other studies,^71^, ^72^, ^73^ includes the use of connectives,^16^ type‐token ratio (TTR as in Bambini et al.^44^) indicating lexical diversity and cohesion in discourse,^74^ givenness^75^ (a measure of contextual information the speaker assumes is already known to listener) to reflect semantic cohesion. These variables refer to explicit cues in speech allowing listeners to make connections between various elements of a speaker's narrative,^76^ and thus reflect the production of pragmatic features in speech. These variables were extracted with the tool for the automatic analysis of cohesion^76^ (TAACO 2.0.4). The tool incorporates a part‐of‐speech (POS) tagger from the natural language tool kit^77^ and synonym sets from the WordNet lexical database.^78^

Our variable selection is in line with a recent overarching review^79^ that highlights altered lexico‐semantics, cohesive production pragmatics and complex syntax in schizophrenia. The actual variable set from the various dictionary‐based and corpus linguistic tools was selected based on the following inclusion criteria: (1) represents the main variable of an index; (2) does not significantly overlap with other variables within the index or tool; (3) captures large amounts of information, independent of other measures; and (4) uses linguistic markers that have been validated in previous work. This resulted in a total of 12 variables included in the analysis. For all indices, the component scores are computed based on standardized scores involving detailed co‐occurrence patterns and the frequencies of each variable.

Following transcription of the interview to text, the language data was then analyzed with three NLP tools to extract these 12 variables and transformed into 12 quantitative scores for each participant (see Supplemental Materials for how each variable was calculated).

### 2.5 Statistical analysis

#### 2.5.1 Cluster analysis

These 12 speech variables were then analyzed through a hierarchical cluster analysis conducted in R.^80^ This unsupervised analysis (i.e., without using any diagnostic or class labels) included all 147 subjects and their 12 speech variables. All data was then standardized to make variables comparable. The agglomerative coefficient was then calculated for four different clustering methods (average, single, complete, and ward), wherein the highest coefficient (closest to 1) suggests the strongest clustering solution.

**FIGURE 1:**
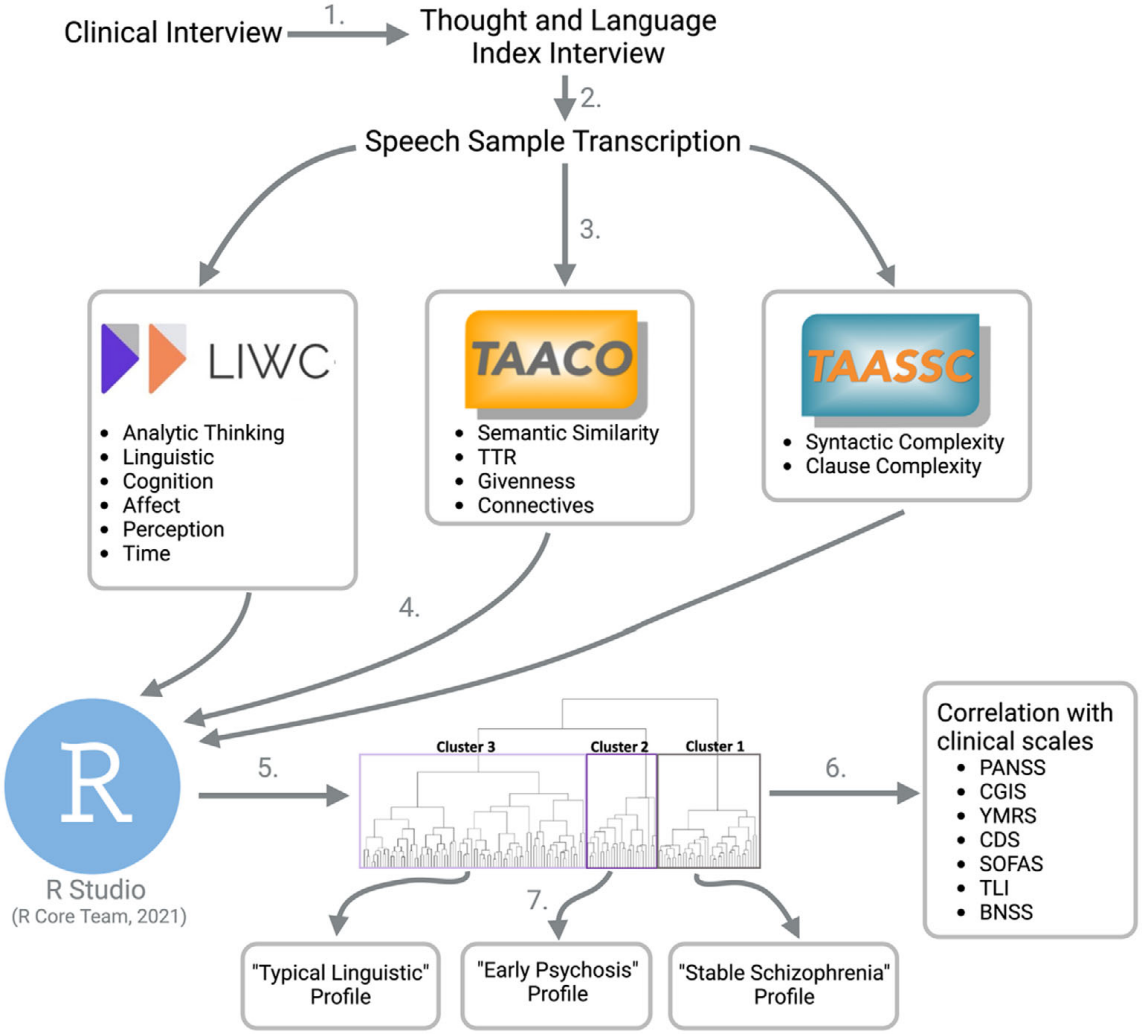
Visual representation of the methodology. Clinical interviews conducted by psychiatrists to determine diagnosis. (1) follow‐up with research assistants for interview to assess cognition, including the Thought and Language Index; (2) Speech data obtained during interview transcribed to text; (3) Speech data analyzed by three different language processors to determine individual scores on each of the 12 linguistic variables; (4) Cluster analysis conducted to determine if there were clusters of individuals with similar linguistic profiles based on the extracted variables; (5) Visual representation of the optimal clustering solution according to Nbclust; (6) Clusters then correlated with clinical scales to assess the relationship between linguistic profiles and clinical symptomatology; (7) Each cluster's linguistic and clinical characteristics were then analyzed to determine any overarching themes among each group.

Nbclust,^81^ which is an R package used to determine the best number of clusters from a dataset through analysis with 30 indices including varying combinations of number of clusters, distance measures and clustering methods, was then used to analyze the data for the optimal number of clusters. Nbclust allows for the validation of the optimal number of clusters by simultaneously evaluating several cluster schemes to help determine which clustering method is the most appropriate. This package provides the user with several methods to determine the optimal number of clusters, including graphical methods (Hubert and D index) in addition to a comprehensive breakdown of the optimal number of clusters determined with different methods. Once the optimal number of clusters was determined, subjects were then divided into their subgroups and the cluster participant composition and cluster‐specific variables were compared between groups to determine any notable features (e.g.,  a disproportionate number of diagnostic categories present in a cluster, or significant differences in the distribution of speech variables among the clusters) to assess linguistic subgroups. Once determined, a dendrogram (seen in Figure 1) was generated which represented the cluster solution, providing a visual representation of the hierarchical solution.

#### 2.5.2 Group analysis

Following cluster analysis, a Pearson Chi‐Square test was used to determine the significant differences between the expected and observed number of participants (CHR, SZ, FEP, HC) included in each group. Demographic characteristics between clusters were analyzed to determine if any clusters significantly differed. Mean group values for each of the linguistic variables were analyzed by one‐way ANOVA to determine any significant differences in scores between groups and then corrected with a Bonferroni correction for multiple comparisons. Additionally, clinical scale scores collected during the initial assessment from participants with FEP and SZ were then used to determine the differences between the clusters with a one‐way ANOVA (Bonferroni corrected for multiple comparisons). All analyses were done in SPSS 24, with alpha set at 0.05.

## 3 RESULTS

### 3.1 Participants characteristics

Demographic measures are provided in Table 1. There was a significant difference between clinical groups on age (*F* = 9.716, *p* <0.001) and education (*F* = 5.331, *p* = 0.002), but not gender (*χ* ^2^ = 3.30, *p* = 0.348) or SES (*F* = 0.531, *p* = 0.662). Follow‐up which examined the diagnostic trajectory of the 72 FEP revealed the following diagnoses: 57 Schizophrenia, 4 Schizoaffective, 1 Schizophreniform, 3 Major Depressive Disorder, 5 Psychosis not otherwise specified, and 2 Bipolar type 1.

**TABLE 1:** Demographic information.

	All participants = 147	Healthy controls (*n* = 39)	First episode psychosis (*n* = 72)	Schizophrenia (*n* = 18)	Clinical high risk (*n* = 18)	Statistics
Gender (male/female)	111/35	26/13	59/13	14/4	13/5	*χ* ^2^ = 3.30, *p* = 0.348
Age (M [SD])	22.83 (5.00)	21.79 (3.47)	22.24 (4.37)	28.47 (7.64)	22.00 (3.89)	*F* = 9.716, *p* <0.001
NS‐SEC (M [SD])	3.38 (1.23)	3.19 (1.40)	3.42 (1.25)	3.36 (1.15)	3.69 (0.75)	*F* = 0.531, *p* = 0.662
Education (M [SD])	15.06 (1.98)	16.11 (2.14)	14.61 (1.73)	14.76 (2.28)	14.89 (1.53)	*F* = 5.331, *p* = 0.002
% Medicated with antipsychotics	37.41%	0	51.4%	100%	0	
Mean DDD (SD)	N/A	0	0.34 (0.57)	1.45 (0.72)	0	
PANSS‐8 Total (M [SD])	22.29 (9.26)	N/A	24.41 (8.88)	14.12 (7.52)	N/A	
PANSS‐8 Positive (M [SD])	10.87 (4.14)	N/A	11.90 (3.95)	6.88 (3.50)	N/A	
PANSS‐8 Negative (M [SD])	6.57 (4.31)	N/A	7.20 (4.45)	7.12 (4.86)	N/A	
TLI Total	2.22 (2.37)	0.62 (0.79)	3.17 (2.76)	1.41 (2.37)	N/A	
Cluster 1	37	12 (30%)	16 (22%)	6 (33%)	3 (17%)	
Cluster 2	27	2^a^ (5%)	21^a^ (29%)	0^a^ (0%)	4 (22%)	
Cluster 3	83	25 (64%)	35 (49%)	12 (67%)	11 (61%)	

*Note*: Education classified as years in school (i.e., completed high school = 14; completed college = 16; completed university = 18). % Medicated includes the proportion of participants on antipsychotics at time of interview. This includes SCZ and FEP. DDD = Daily Defined Dose which is calculated for FEP.

Abbreviations: M, mean; NS‐SEC, national statistics socio‐economic classification in parents; SD, standard deviation.

^a^ A significant difference between expected and observed numbers. All participant values for PANSS‐8 Total, PANSS Positive, PANSS Negative, represent SCZ and FEP participants only. TLI – Thought and Language Index Cluster 1; Stable Phase Profile. Cluster 2; Early Phase Profile. Cluster 3; Typical Linguistic Profile. Note that 2 out of the 18 CHR subjects developed FEP in the 2‐year follow‐up.

### 3.2 Cluster analysis

The agglomerative coefficient determined that Ward's method was the strongest clustering solution (0.90) among the four methods (Ward, 0.90; Complete, 0.79; Average, 0.72; Single, 0.66). Nbclust suggested three clusters as the optimal clustering solution from the sample based on a simulation of potential clustering solutions (Simulation of 23 cluster analyses proposed 11 solutions with 3 clusters, and 6 with 2 clusters). In line with our prior clustering studies,^67^, ^82^ this clustering solution was further validated by the Hubert and D‐index.

Cluster 1 consisted of 37 participants (3 CHR, 6 SZ, 16 FEP, 12 HC), cluster 2 consisted of 27 participants (4 CHR, 0 SZ, 21 FEP, 2 HC), and cluster 3 consisted of 83 participants (11 CHR, 12 SZ, 35 FEP, 25 HC). Cluster 3 comprised of the majority of participants from each clinical group and HCs [48%–67% of each group], thus representing the most typical linguistic profile irrespective of diagnostic status. Of the 2 atypical subgroups, one‐third of SZ were in cluster 1, making this the only atypical cluster with later stage of psychosis patients (i.e., SZ) included. Cluster 2 captured most of the ‘atypical’ FEP and CHR subjects (22%–29%), with no SZ patients and just 2 healthy subjects in this cluster. Pearson Chi‐Square test was significant for group membership differences between groups (*χ* ^2^ = 15.0632, *df* = 6, *p* = 0.020), with cluster 2 having a disproportionately greater amount of FEP and a disproportionately lower amount of SZ and HC compared to the other two subgroups. Thus, cluster 1's linguistic pattern represents more stable pattern of illness seen in later stages, while cluster 2 consisted primarily of an early‐stage illness profile. In terms of demographics, there was no significant difference between clusters on education (*F* = 0.194, *p* = 0.824), SES (*F* = 0.273, *p* = 0.761), age (*F* = 0.418, *p* = 0.659), or gender (*χ* ^2^ = 0.430, *p* = 0.807). No significant differences between cluster 1 and 2 regarding the proportion of FEP who were later diagnosed with schizophrenia in comparison to other diagnoses (*χ* ^2^ = 0.133, *p* = 0.715).

### 3.3 Linguistic variables

A one‐way ANOVA was performed to compare the three clusters on the 12 linguistic variables (Table 2). The one‐way ANOVA revealed a statistically significant difference between at least two groups on all 12 variables (*p* ≤ 0.001 on all variables, except ‘time’ which had *p* = 0.038). Pairwise comparisons with a Bonferroni correction revealed significant differences among all 3 clusters for Analytic Thinking, words referring to Linguistic Function and Perception, Clause Complexity, Givenness, and Type‐Token Ratio. Additionally, significant differences were noted between 2 clusters for words referring to Cognition, Affect and Time, Syntactic Complexity, Semantic Similarity, and Connectives (Table 2).

**TABLE 2:** Pairwise comparisons for linguistic variables between groups.

	Between group differences	Cluster 1 (*n* = 37)	Cluster 2 (*n* = 27)	Cluster 3 (*n* = 83)	Group differences	Interpretation
Analytic thinking	*F* = 54.49, *p* <0.001	75.9 ± 2.18	29.1 ± 2.95	53.1 ± 2.2	1 >3 >2	High in SPP; low in EPP
Linguistic	*F* = 75.79, *p* <0.001	75.2 ± 0.508	83.9 ± 0.521	80.4 ± 0.314	2 >3 >1	High in EPP; Low in SPP
Cognition	*F* = 37.91, *p* <0.001	7.53 ± 0.41	13.7 ± 0.853	13.1 ± 0.384	2,3 >1	Low in SPP
Affect	*F* = 7.91, *p* = 0.001	1.06 ± 0.108	2.06 ± 0.236	1.73 ± 0.125	2,3 >1	Low in SPP
Perception	*F* = 53.04, *p* <0.001	17.7 ± 0.334	11.8 ± 0.403	14.2 ± 0.282	1 >3 >2	High in SPP; Low in EPP
Time	*F* = 3.358 *p* = 0.038	0.943 ± 0.15	1.5 ± 0.193	1.15 ± 0.0843	2 >1	High in EPP, low in SPP
Syntactic complexity	*F* = 8.18, *p* <0.001	40.2 ± 0.985	47.7 ± 1.46	46 ± 1.02	2,3 >1	low in SPP
Clause complexity	*F* = 29.79, *p* <0.001	6.39 ± 0.082	7.39 ± 0.0868	7 ± 0.0624	2 >3 >1	High in EPP; Low in SPP
Semantic similarity	*F* = 8.28, *p* <0.001	1.09 ± 0.0108	1.16 ± 0.0261	1.07 ± 0.0109	2 >1,3	High in EPP
Givenness	*F* = 43.51, *p* <0.001	0.949 ± 0.0261	1.48 ± 0.0566	1.1 ± 0.0256	2 >3 >1	High in EPP; Low in SPP
All connectives	*F* = 37.45, *p* <0.001	0.0501 ± 0.00322	0.09 ± 0.00459	0.0599 ± 0.00182	2 >1,3	High in EPP
Type‐token ratio (TTR)	*F* = 7.13, *p* = 0.001	0.627 ± 0.00998	0.613 ± 0.01	0.66 ± 0.00726	3 >1 >2	Low in EPP and SPP

*Note*: Between group differences represents a one‐way ANOVA (*df* = 2144 for entire sample). Values are means ± SEM. Significant group differences (*p* <0.05) are outlined in the final column, where “>” indicates a significant difference between groups as analyzed by one‐way ANOVA and Bonferroni correction for multiple comparisons. The atypical language profile seen in early psychosis (EPP; cluster 2) has low ATI, more linguistic and connective words with higher complexity, similarity, higher givenness, low perception. The atypical profile seen in the stable phase (SPP; cluster 3), has higher ATI, low cognition, affect but high perception words with lower complexity (especially in subordination and length of production), and givenness. Both atypical profiles are characterized by lower TTR.

As Syntactic Complexity was the only summed item wherein cluster 2 did not differ from cluster 3, we further analyzed the 5 component subscales (Type 1 to Type 5; see Table 3) to explore the subitems showing  group differences among the clusters. A one‐way ANOVA revealed statistically significant differences among at least two groups on Type 1 (*p* = 0.002), Type 2 (*p* <0.001), Type 3 (*p* <0.001), Type 4 (*p* <0.001), but not Type 5 (*p* = 0.084). Group differences are reported in Table 1 based on a pairwise comparison with a Bonferroni correction.

**TABLE 3:** Syntactic complexity subscales.

Subscale name	Subscale calculation	Group differences
Type 1. Length of production units	Mean length of sentence, mean length of T‐unit, mean length of clauses.	2,3 >1
Type 2. Sentence complexity	Clauses per sentence.	2 >3 >1
Type 3. Amount of subordination	Complex T‐unit ratio, clauses per T‐unit, dependent clauses per clause, dependent clauses per T‐unit.	2,3 >1
Type 4. Amount of coordination	T units per sentence, coordinate phrases per T‐unit, coordinate phrases per clause.	2 >1,3
Type 5. Particular syntactic structure and larger production units	Verb phrases per T‐unit, complex nominals per T‐unit, complex nominals per clause.	n.s.

*Note*: Type 1–3 scores were low in the atypical cluster that had patients with established schizophrenia, while type 2 and 4 scores were higher in the atypical cluster with early stage patients.

### 3.4 Clinical scales

Clinical scores on symptoms from FEP and SZ participants (*n* = 90; 72 FEP and 18 SZ) were compared among the 3 clusters. Cluster 1 consisted of 22 participants (16 FEP and 6 SZ), cluster 2 consisted of 21 participants (21 FEP and 0 SZ), and cluster 3 consisted of 47 participants (35 FEP and 12 SZ). A one‐way ANOVA comparing the three clusters on the seven clinical scales revealed a statistically significant difference between at least two clusters for PANSS Disorganization, PANSS General, YMRS, TLI Overall, TLI Impoverishment, and TLI Disorganization (Table 4). Pairwise comparison with a Bonferroni correction determined significant differences among all three clusters for TLI total scores (cluster 2 higher than 3 and 1), and a consistently higher score for cluster 2 for PANSS Disorganization, PANSS General, YMRS, TLI Impoverishment, TLI Disorganization (Table 4). For all significant differences in clinical rating scales, cluster two scored higher than other clusters.

**TABLE 4:** Pairwise comparisons for clinical scales.

	Between group differences	Cluster 1 (16 FEP, 6 SZ)	Cluster 2 (21 FEP, 0 SZ)	Cluster 3 (35 FEP, 12 SZ)	Group differences	Interpretation
FEP diagnosis at 6 months	*χ* ^2^ = 8.461, *p* = 0.574	13 SZ, 1 BP, 1 PNOS, 1 MDD	18 SZ, 2 SZA, 1 BP	26 SZ, 2 SZA, 4 PNOS, 2 MDD, 1 SZP	n.s	
PANSS‐8 total	*F* = 1.387, *p* = 0.256	22.61 ± 2.025	26.05 ± 1.764	22.64 ± 1.173	n.s.	
PANSS positive	*F* = 2.449, *p* = 0.093	10.39 ± 0.793	12.85 ± 0.862	10.98 ± 0.57	n.s.	
PANSS negative	*F* = 0.639, *p* = 0.531	7.89 ± 1.099	6.85 ± 0.985	6.57 ± 0.586	n.s	
PANSS disorganization	*F* = 4.020, *p* = 0.022	3.44 ± 0.39	5.7 ± 0.692	4.34 ± 0.372	2 >1	High in EPP
PANSS general	*F* = 4.182, *p* = 0.019	4.22 ± 0.432	6.35 ± 0.534	5.09 ± 0.365	2 >1	High in EPP
CGIS	*F* = 1.243, *p* = 0.295	4.82 ± 0.312	5.4 ± 0.285	4.83 ± 0.234	n.s.	
YMRS	*F* = 6.942, *p* = 0.002	7.27 ± 1.624	15.81 ± 2.081	9.34 ± 1.073	2 >1,3	High in EPP
CDS	*F* = 0.140, *p* = 0.870	3.47 ± 0.777	3.45 ± 0.651	3.07 ± 0.532	n.s.	
SOFAS	*F* = 1.944, *p* = 0.149	46.95 ± 2.753	38.76 ± 2.267	43.62 ± 2.225	n.s.	
TLI overall	*F* = 11.075, *p* <0.001	0.460 ± 0.010	1.587 ± 0.240	0.87 ± 0.112	2 >3 >1	High in EPP, low in SPP
TLI impoverishment	*F* = 3.430, *p* = 0.037	0.238 ± 0.073	0.563 ± 0.109	0.319 ± 0.063	2 >1	High in EPP, low in SPP
TLI disorganization	*F* = 6.766, *p* = 0.002	0.222 ± 0.072	1.024 ± 0.222	0.553 ± 0.0990	2 >1,3	High in EPP
BNSS total	*F* = 1.900, *p* = 0.157	20.44 ± 3.796	28.31 ± 4.901	18.47 ± 2.574	n.s.	

*Note*: Between group differences represents a one‐way ANOVA (*df* ranges from 2, 74–87 as a result of incomplete clinical scales by some participants). Values are means ± SEM. Values are means ± SEM. Significant group differences (*p* <0.05) are outlined in the final column, where “>” indicates a significant difference (and “n.s.” for non‐significant) between groups as analyzed by one‐way ANOVA and Bonferroni correction for multiple comparisons. The clinical scales only included FEP (*n* = 72) and SZ (*n* = 18). The symptom profile seen in early psychosis (EPP; cluster 2) is characterized by higher YMRS scores and higher disorganization when compared to the stable phase profile (SPP; cluster 3). SZA, Schizoaffective, SZP, Schizophreniform, BP, Bipolar, MDD, Major Depressive Disorder, PNOS, Psychosis Not Otherwise Specified, BPE, Brief Psychotic Episode. Brackets in the Cluster x FEP cells represents the diagnostic breakdown of FEP based on 6‐month follow‐up. disorganization and impoverishment but did not differ from the other patients in level of functioning.

## 4 DISCUSSION

Linguistic analysis of speech profile has a long history in the study of ‘formal thought disorder’ in schizophrenia.^32^, ^83^, ^84^ Through the use of speech technology (NLP) based digital subtyping analysis we were able to investigate healthy and pathological variations in lexico‐semantic, syntactic and pragmatic features in discourse within a broad sample. This work allows us to report three key findings. (1) There is a systematic variation in linguistic profiles among patients with psychosis; this results in an identifiable number of subgroups with distinct linguistic profiles. (2) Most patients (49%–67%) clustered along with 64% of HCs forming a ‘typical language subgroup’ (Cluster 3). Furthermore, only 33%–51% of patients had an atypical language profile (Clusters 1 and 2). (3) One of the two atypical subgroups had very low number of healthy subjects in addition to no individuals with established schizophrenia (Cluster 2). This subgroup has a profile exclusive to early stages of subthreshold (CHR) or ‘suprathreshold’ (FEP) psychosis, with higher load of clinically detectable disorganization and impoverishment among the FEP. Taken together, at least two forms of linguistic deviation occur in psychotic disorders, one of which is restricted to early stages of psychosis.

These distinct linguistic profiles among the clusters allowed us to further characterize them. Cluster 1 represents a stable phase schizophrenia profile (SPP; 31% HC) which includes 33% of the SZ sample, in addition to a lower proportion of FEP (22% vs. the 29%–48% in the other samples). Though in terms of absolute numbers, most subjects in this cluster 1 are FEP (*n* = 16), members of this group showed less pronounced deviations in speech and lower illness burden than the other atypical cluster – cluster 2. Cluster 2 represents the early phase psychosis profile (EPP; 5% HC), consisting almost entirely of FEP (77.8% of cluster) with no SZ. This cluster's deviations in speech, was accompanied by higher disease burden when compared to the other clusters. As a result of this cluster largely consisting of FEP, this cluster provides support for the presence of distinct linguistic disturbances during the early disease course. Lastly, cluster 3 represents the typical language profile (TLP; 64% HC), which consists of the largest proportion of HCs in addition to experiencing less deviations in speech when compared to the other clusters.

In our sample, cluster 2 consisted of a disproportionately high amount of FEP individuals (77.77% of the cluster compared to 48.98% overall sample) and a disproportionately low amount of SZ and HC when compared to the other clusters (0% and 7.4% compared to 12.2% SZ and 26.5% HC, respectively in the overall sample). FEP subjects in this cluster exhibited higher burden of formal thought disorder – both disorganization and impoverishment from TLI and PANSS conceptual disorganization scores. In terms of linguistic variables, members of this cluster had lower Analytical Thinking indicating less logical organization of thoughts. Cluster 2 subjects also had more pronounced use of the “linguistic class” words (including personal pronouns, conjunctions, modals and auxiliary verbs) and connectives but lower expression of words reflecting “perception” (e.g., in, out, up, there, to the left). This pattern may produce more coordinated clauses, for example through repeated use of connectives, resulting in complex constructions that are not particularly descriptive of the picture content. In fact, there was a higher degree of syntactic complexity and semantic similarity in their speech, both of which has been noted in prior studies of early stage psychosis.^13^, ^71^, ^85^ Notably, while semantic similarity may not change during the illness course,^71^ syntactic complexity may notably reduce over time especially when the diagnosis of schizophrenia gets established.^12^, ^13^, ^86^, ^87^

The higher givenness scores in cluster 2 subjects indicate a more frequent assumption that the listener has some pertinent information about the content that they are referring to, resonating with studies that indicate a failure of Theory of Mind especially in acute phases of psychosis with higher degree of disorganization.^88^, ^89^ Notably, none of the established schizophrenia patients and a very small number of healthy subjects had the linguistic profile seen in cluster 2 subjects, indicating the importance of specifying stage of illness when studying speech‐based NLP features in psychiatric disorders.

The speakers' tendency to repeat syntactic forms and use the same semantic content of the prime (a picture) indicates perseveration in speech, a well‐known feature of schizophrenia.^90^ Both cluster 1 and cluster 2 subjects scored significantly lower on TTR than the more typical cluster 3, suggesting that the deficits in this domain of speech may persist even in later phases of a treated psychotic illness. Lower TTR is a well replicated observation in schizophrenia.^91^ Low TTR may indicate lexical repetitiveness (reflected in smaller connectomes in word graph studies^92^ or the recourse to the same syntactic structure during a discourse). The latter, called structural priming,^93^ may result from preceding syntactic context influencing the subsequent ones,^94^ reflecting context processing errors in psychosis. Recurrence of same entities,^6^ including higher use of pronouns^95^ can also contribute to lower TTR in patients observed here.

Cluster 1 subjects included both SZ, early‐stage patients and a proportion of healthy subjects, indicating that the linguistic profile seen among cluster 1 members can be seen as a variation that is not still fully dissociated from the healthy range. Cluster 1 subjects had higher analytical thinking but used less words reflecting cognitive and affect processes but more perception words. They had lower complexity (especially in subordination and length of production) and givenness than expected (compared to cluster 3). Low givenness means, in contrast from cluster 2, subjects in cluster 1 spoke as if the information they conveyed had a degree of ‘newness’ that was not present, thus resulting in an atypical information structure in their discourse. In these aspects, and in reference to the typical cluster 3, cluster 1 appeared to be somewhat an inverse of cluster 2. Importantly, these two profiles did not map on positive and negative thought disorder (i.e., impoverishment and disorganization syndromes); instead, they varied with illness stages in our sample. Thus, the atypical linguistic profile in later stages (cluster 1 profile) is not a mere quantitative variation of the profile seen in early stages (cluster 2 features), but rather a distinct picture that is associated with lower burden of overall illness severity, especially less prominent thought disorder and affective features.

### 4.1 Limitations/Future work

Our work has several strengths including patients with a spectrum of severity from pre‐psychotic to later stages, with many untreated patients, leveraging the variation among healthy subjects^46^ and using multi‐level linguistic analysis that included lexico‐semantic, pragmatic and syntactic variables. Nevertheless, our cross‐sectional design limits investigating the stability of reported subgroups. While our sample size was sufficiently powered to detect a cluster structure and was larger than most other clustering studies employing similar variable sets,^44^, ^96^ we had a disproportionate number of FEP compared to CHR and SZ subjects. This had a likely influence on both atypical clusters having more FEP subjects, including in cluster 1 with a more stable phase symptom profile (low overall symptom burden and disorganization). As a result, we do not interpret cluster 1 as a ‘chronic illness’ profile; but merely a profile that is likely to continue beyond the prodrome and first episode, even when symptom burden is stably low. Future work including the inclusion of more SZ and CHR subjects would help further elucidate variations in later stages of illness and increase the generalizability of these findings. Additionally, aberrations in everyday language use are known to be ‘paroxysmal’, especially in otherwise clinically stable subjects; the lack of a symptom provocation approach and more than one time point of assessment might have reduced our ability to detect atypical linguistic profiles. Our estimate that 33% of patients with schizophrenia display an atypical language profile is best considered to be conservative. Finally, we did not use a conversation analysis; the interviewer prompts were minimal and used only when there was notable poverty of speech.

To conclude, we provide preliminary evidence for a distinct linguistic profile in the early stages of psychosis. A second and distinct linguistic profile, also seen in the early stages, was not specific to this period, as it is seen in several healthy control subjects and patients with established schizophrenia. This observation raises the question of whether two (or more) distinct pathophysiological processes affect everyday language in psychosis. One of these processes may be more florid, giving rise to an early‐stage profile, while the other is likely more persistent, affecting long‐term outcomes. In terms of clinical practice, this work provides an early demonstration of how digitally recorded 1‐min speech samples can help identify distinct linguistic profiles in early‐phase psychosis, that map onto symptom severity. These linguistic profiles likely indicate the underlying ‘formal thought disorder’ and are clinically apparent when speech is analyzed at the lexical, syntactic and semantic levels. This work also has implications for sampling strategies in future speech NLP studies in psychosis, an expanding area of digital psychiatry.^35^

## AUTHOR CONTRIBUTIONS

Lena Palaniyappan planned and designed the study and supervised the work of all the other authors. Michael Mackinley recruited patients and collected the data. Liangbing Liang and Tyler C. Dalal designed and conducted the statistical analyses. Angelica M. Silva applied the linguistic tools to the transcribed data, analyzed and interpreted the linguistic profiles. Tyler C. Dalal, Angelica M. Silva, and Alban Voppel created the original draft. All authors contributed to the final manuscript.

## FUNDING INFORMATION

The TOPSY study was funded by the Canadian Institutes of Health Research (CIHR) Foundation Grant (Grant no. 375104/2017) to Lena Palaniyappan with data acquisition supported by the Canada First Excellence Research Fund to BrainSCAN, Western University (Imaging Core); Innovation fund for Academic Medical Organization of Southwest Ontario (PROSPECT study). Compute Canada Resources (Application No. 1530) were used in the storage and analysis of imaging data. Lena Palaniyappan acknowledges research support from the Canada First Research Excellence Fund, awarded to the Healthy Brains, Healthy Lives initiative at McGill University (through New Investigator Supplement to Lena Palaniyappan); Monique H. Bourgeois Chair in Developmental Disorders and Graham Boeckh Foundation (Douglas Research Centre, McGill University) and salary award from the Fonds de recherche du Quebec‐Santé (FRQS). Liangbing Liang was funded by a studentship from the Canada First Excellence Research Fund to BrainSCAN, Western University. Angelica M. Silva's position was funded by a Project Grant from the Canadian Institutes of Health Research (Grant no. FRN 391348). SF was partly supported by a grant from the Children's Health Foundation, London, ON to the PROSPECT clinic at the Programme for Early Intervention and Prevention in Psychosis (PEPP London). Tyler C. Dalal was supported by Chrysalis Foundation (London Ontario), Alban Voppel is partly supported by a Wellcome Trust (Palaniyappan/Sommer: 226168/Z/22).

## CONFLICT OF INTEREST STATEMENT

Lena Palaniyappan reports personal fees for serving as chief editor from the Canadian Medical Association Journals (the JPN), speaker/consultant fees from Janssen Canada and Otsuka Canada, SPMM Course Limited, UK, Canadian Psychiatric Association; book royalties from Oxford University Press; investigator‐initiated educational grants from Janssen Canada, Sunovion and Otsuka Canada outside the submitted work. All other authors report no potential conflicts.

### PEER REVIEW

The peer review history for this article is available at https://www.webofscience.com/api/gateway/wos/peer‐review/10.1111/acps.13685.

## ETHICS STATEMENT

The studies involving human participants were reviewed and approved by the Western University Health Sciences Research Ethics Board. The patients/participants provided their written informed consent to participate in this study.

## Supporting information


**Data S1:** Supporting Information.

## Data Availability

The authors will be making the anonymised transcripts of the data used in the study available through the DISCOURSE in Psychosis consortium’s Psychosis Talkbank https://psychosis.talkbank.org/. Requests to access the datasets should follow the instructions on this website.
