# Supplementary material for: Speech based natural language profile before, during and after the onset of psychosis: A cluster analysis
Source: Acta Psychiatr Scand. 2024 Apr 10;151(3):332–47. doi: 10.1111/acps.13685 (PMC11787926; doi:10.1111/acps.13685)
Supplement: Supplementary file 1 — Data S1: Supporting Information. [file ACPS-151-332-s001.pdf]

Supplemental Materials

Language Variables

3 NLP tools included in analysis – LIWC-22, TAACO-2.0.4, and TAASC-1.3.8. Indices included in analysis were chosen if they were the main variable of an index and did not overlap significantly with other variables within the index. For example, content_ttr index looks at the number of unique content words (types) divided by the number of total content words lemmas (tokens) and is made up of the noun_ttr, verbs_ttr, adjectives_ttr, and adverbs_ttr indices. Therefore, since content_ttr encapsulates these 4 other index domains, we chose to only include the primary index (content_ttr) to avoid high levels of similarity between measures, while still capturing a large amount of information.

In total, 12 indices which represented 12 variables were included in the analysis. Lexical variables included Analytic Thinking, Linguistic, Cognition, Affect, Perception, and Time and were analyzed with LIWC-22 (Boyd et al., 2022). Syntactic variables included Syntactic Complexity and Clause Complexity and were analyzed with TAASC 1.3.8 (TAASSC 1.3.8.; Kyle, 2016; Lu, 2010). Semantic cohesion variables included semantic similarity, Type-Token Ratio (TTR), Givenness, and Connectives and were analyzed with TAACO 2.0.4 Crossley et al., 2016). All speech processing tools provided quantitative values for each of the variables listed.

**Speech Domain**	**Tool**	**Variables**
Lexical	LIWC-22	Analytic thinking, linguistic, cognition, affect, perception, time
Syntax	TAASSC 1.3.8 (open source)	Syntactic complexity, clause complexity
Semantic Cohesion	TAACO 2.0.4 (open source)	Semantic similarity, Type-Token ratio (TTR), Givenness, Connectives

1. **Lexical Variables**

Note: all lexical variables are expressed as percentage of total words used in any given language sample except for total word count, words per sentence. The four summary variables (analytic, clout, authentic and tone) are converted to percentiles based on standardized scores from a large comparison corpora.

The adapted table on the following page only includes the variables included in our analysis. For a full list of the linguistic variables available see https://www.liwc.app/.

Adapted Table 2 from The Development and Psychometric Properties of LIWC-22

Category	Abbrev.	Description/Most frequently used exemplars	Words/ Entries in category*	Internal Consistency: Cronbach's α	Internal Consistency: KR-20
**1. Analytical thinking**	Analytic	Metric of logical, formal thinking	-	-	-
Articles	article	a, an, the, alot	-	-	-
Prepositions	prep	to, of, in, for	-	-	-
Personal Pronouns	ppron	I, you, my, me	-	-	-
Impersonal Pronouns	iprom	that, it, this, what	-	-	-
Auxillary verbs	auxverb	is, was, be, have	-	-	-
Adverbs	adverb	so, just, about, there	-	-	-
Conjunctions	conj	and, but, so, as	-	-	-
Negations	negate	not, no, never, nothing	-	-	-
**2. Linguistic**	Linguistic		4933	0.36	1.00
Total function words	function	the, to, and, I	499/1443	0.28	0.99
Total pronouns	pronoun	I, you, that, it	74/286	0.43	0.97
Personal pronouns	ppron	I, you, my, me	42/221	0.24	0.95
1st person singular	i	I, me, my, myself	6/74	0.49	0.85
1st person plural	we	we, our, us, lets	7/17	0.43	0.78
2nd person	you	you, your, u, yourself	14/59	0.37	0.82
3rd person singular	shehe	he, she, her, his	8/30	0.58	0.83
3rd person plural	they	they, their, them, themsel*	7/20	0.36	0.69
Impersonal pronouns	ipron	that, it, this, what	32/68	0.43	0.91
Determiners	det	the, at, that, my	97/293	-0.19	0.95
Articles	article	a, an, the, alot	3/103	0.12	0.61
Numbers	number	one, two, first, once	44/61	0.57	0.87
Prepositions	prep	to, of, in, for	83/302	0.16	0.95
Auxiliary verbs	auxverb	is, was, be, have	25/282	0.44	0.97
Adverbs	adverb	so, just, about, there	159/514	0.63	0.97
Conjunctions	conj	and, but, so, as	49/65	0.11	0.89
Negations	negate	not, no, never, nothing	8/247	0.49	0.92
Common verbs	verb	is, was, be, have	1560	0.60	0.99
Common adjectives	adj	more, very, other, new	1507	0.26	0.99
Quantities	quantity	all, one, more, some	422	0.45	0.96
**3. Cognition**	Cognition	is, was, but, are	1403	0.68	0.99
All-or-none	allnone	all, no, never, always	35	0.37	0.88
Cognitive processes	cogproc	but, not, if, or, know	1365	0.67	0.99
Insight	insight	know, how, think, feel	383	0.43	0.96
Causation	cause	how, because, make, why	169	0.21	0.90
Discrepancy	discrep	would, can, want, could	108	0.29	0.91
Tentative	tentat	if, or, any, something	230	0.52	0.94
Certitude	certitude	really, actually, of course, real	131	0.22	0.88
Differentiation	differ	but, not, if, or	325	0.38	0.94
Memory	memory	remember, forget, remind, forgot	26	0.23	0.64
**4. Affect**	Affect	good, well, new, love	2999	0.64	0.99
Positive tone	tone_pos	good, well, new, love	1020	0.61	0.98
Negative tone	tone_neg	bad, wrong, too much, hate	1530	0.62	0.98
Emotion	emotion	good, love, happy, hope	1030	0.61	0.97
Positive emotion	emo_pos	good, love, happy, hope	337	0.52	0.93
Negative emotion	emo_neg	bad, hate, hurt, tired	618	0.52	0.95
Anxiety	emo_anx	worry, fear, afraid, nervous	120	0.37	0.80
Anger	emo_anger	hate, mad, angry, frustr*	181	0.30	0.82
Sadness	emo_sad	:(, sad, disappoint*, cry	134	0.25	0.80
Swear words	swear	shit, fuckin*, fuck, damn	462	0.79	0.93
**5. Perception**	Perception	in, out, up, there	1834	0.59	0.99
Attention	attention	look, look* for, watch, check	130	0.16	0.86
Motion	motion	go, come, went, came	485	0.42	0.97
Space	space	in, out, up, there	617	0.41	0.98
Visual	visual	see, look, eye*, saw	226	0.49	0.94
Auditory	auditory	sound*, heard, hear, music	255	0.49	0.91
Feeling	feeling	feel, hard, cool, felt	157	0.32	0.90
**6. Time orientation**					
Time	time	when, now, then, day	464	0.50	0.97
Past focus	focuspast	was, had, were, been	699	0.71	0.98
Present focus	focuspresent	is, are, I'm, can	373	0.60	0.96

2. Semantic Variables

Semantic variables include Connectives, Givenness, Type-Token Ratio, Semantic Similarity.

2.1 Connectives

The connectives index is made up of 25 indices. All_connectives were chosen for our analysis since it was the primary index and included all the connectives in text.

Index Name	In text name	Description	Denominator	Examples
all_connective	all connectives	number of all connectives	number of words in text	actually, admittedly, after

2.2 Givenness

Givenness index is the sum of the 4 indices included in the table.

Index Name	In text name	Calculation Method
pronoun_density	pronoun density	number of third person pronouns divided by number of words
pronoun_noun_ratio	pronoun to noun ratio	number of third person pronouns divided by number of nouns
repeated_content_lemmas	repeated content lemmas	number of repeated content lemmas divided by number of words
repeated_content_and_pronoun_lemmas	repeated content lemmas and pronouns	number of repeated content and third person pronouns divided by number of words

2.3 Type-Token ratio (TTR)

TTR indices included: content_ttr, function_ttr.

Index Name	In text name	Calculation Method
content_ttr	content lemma TTR	number of unique content word lemmas (types) divided by the number of total content word lemmas (tokens).<br>Content = nouns, verbs, adjectives, adverbs
function_ttr	function lemma TTR	number of unique function word lemmas (types) divided by the number of total function word lemmas (tokens).<br>Function = articles, pronouns, appositions, conjunctions, subordinating conjunctions, auxiliary verbs, particles, interjections, expletives, pro-sentences.

2.4 Lexical Overlap (sentence)

Lexical overlap measures the amount of “overlap” of lemmas from one sentence to the next. TAACO outputs lexical overlap for both sentence and paragraph level. We chose to only include sentence because the speech examined was from 1-minute trials and therefore wouldn’t provide enough text for multiple paragraphs.

Index Name	In text name	Index description	Denominator
adjacent_overlap_all_sent	adjacent sentence overlap all lemmas	number of lemma types that occur at least once in the next sentence	number of types in each sentence (except the last sentence)

adjacent_overlap_all_sent_div_seg	adjacent sentence overlap all lemmas (sentence normed)	number of lemma types that occur at least once in the next sentence	number of sentences in text (except last sentence)

2.5 Semantic Similarity

Looks at semantic similarity between sentences.

**Index Name**	**In text name**	**Index description**	**More info**
lsa_1_all_sent	lsa cosine similarity (adjacent sentences)	Average latent semantic analysis cosine similarity between all adjacent sentences.	LSA models trained on the newspaper and magazine sections of the Corpus of Contemporary American English (Davies, 2010)
word2vec_1_all_sent	word2vec similarity (adjacent sentences)	Average word2vec similarity score between all adjacent sentences.	Word2vec models trained on the newspaper and magazine sections of the Corpus of Contemporary American English (Davies, 2010)

3. Syntactic Variables

Examines Syntactic Complexity and Clause Complexity. Syntactic complexity is composed of 5 indices (Types) and Clause Complexity is composed of 31 indices.

3.1 Syntactic Complexity

Syntactic Complexity is composed of 5 indices which are added together to form the overall syntactic complexity score.

Type 1 – length of production units. Composed of MLS, MLT, MLC.

Index Name	SCA Name	Source	Index Type	Description	Numerator	Denominator
av_sent_len	MLS	Syntactic Complexity Analyzer	Unit Length	mean length of sentence	number of words in text	number of sentences in text
av_t_unit_len	MLT	Syntactic Complexity Analyzer	Unit Length	mean length of T-unit	number of words in text	number of T-units in text
av_clause_len	MLC	Syntactic Complexity Analyzer	Unit Length	mean length of clause	number of words in text	number of clauses in text

Type 2 - Sentence complexity. Composed of C/S.

Index Name	SCA Name	Source	Index Type	Description	Numerator	Denominator
av_clause_sent	C/S	Syntactic Complexity Analyzer	Clausal	clauses per sentence	number of clauses in text	number of sentences in text

Type 3 – Amount of subordination – ratios that reflect the amount of subordination. Composed of CT/T, C/T, DC/C, DC/T.

Index Name	SCA Name	Source	Index Type	Description	Numerator	Denominator
perc_complex_tunits	CT/T	Syntactic Complexity Analyzer	Clausal	complex T-unit ratio	number of complex T-units in text	number of T-units in text
av_clause_tunit	C/T	Syntactic Complexity Analyzer	Clausal	clauses per T-unit	number of clauses in text	number of T-units in text
perc_dep_clause	DC/C	Syntactic Complexity Analyzer	Clausal	dependent clauses per clause	number of dependent clauses in text	number of clauses in text
dep_clause_tunit	DC/T	Syntactic Complexity Analyzer	Clausal	dependent clauses per T-unit	number of dependent clauses in text	number of T-units in text

Type 4 – Ratios that measure the amount of coordination. Composed of T/S, CP/T, CP/C.

Index Name	SCA Name	Source	Index Type	Description	Numerator	Denominator
av_tunits_sent	T/S	Syntactic Complexity Analyzer	Clausal	T-units per sentence	number of T-units in text	number of sentences in text
av_coordinate_phrase_tunit	CP/T	Syntactic Complexity Analyzer	Clausal	coordinate phrases per T-unit	number of coordinate phrases	number of T-units in text
av_coordinate_phrase_clause	CP/C	Syntactic Complexity Analyzer	Clausal	coordinate phrases per clause	number of coordinate phrases	number of clauses in text

Type 5 – Particular syntactic structure and larger production units. Composed of VP/T, CN/T, CN/C.

Index Name	SCA Name	Source	Index Type	Description	Numerator	Denominator
av_verb_phrase_tunit	VP/T	Syntactic Complexity Analyzer	Clausal	verb phrases per T-unit	number of verb phrases in text	number of T-units in text
av_complex_nominal_tunit	CN/T	Syntactic Complexity Analyzer	Clausal	complex nominals per T-unit	number of complex nominals	number of T-units in text
av_complex_nominal_clause	CN/C	Syntactic Complexity Analyzer	Clausal	complex nominals per clause	number of complex nominals	number of clauses in text

3.2 Clause Complexity

Composed of 31 indices and then averaged.

1	cl_av_deps	dependents per clause	Clause Complexity
2	cl_ndeps_std_dev	dependents per clause (standard deviation)	Clause Variety
3	acomp_per_cl	adjective complements per clause	Clause Complexity
4	advcl_per_cl	adverbial clauses per clause	Clause Complexity
5	agent_per_cl	passive agents per clause	Clause Complexity
6	cc_per_cl	clausal coordinating conjunctions per clause	Clause Complexity
7	ccomp_per_cl	clausal complements per clause	Clause Complexity
8	conj_per_clause	conjunctions per clause	Clause Complexity

9	csubj_per_cl	clausal subjects per clause	Clause Complexity
10	csubjpass_per_cl	passive clausal subjects per clause	Clause Complexity
11	dep_per_cl	undefined dependents per clause	Clause Complexity
12	discourse_per_cl	discourse markers per clause	Clause Complexity
13	dobj_per_cl	direct objects per clause	Clause Complexity
14	expl_per_cl	existential "there" per clause	Clause Complexity
15	iobj_per_cl	indirect objects per clause	Clause Complexity
16	mark_per_cl	subordinating conjunctions per clause	Clause Complexity
17	ncomp_per_cl	nominal complements per clause	Clause Complexity
18	neg_per_cl	clausal negations per clause	Clause Complexity
19	nsubj_per_cl	nominal subjects per clause	Clause Complexity
20	nsubjpass_per_cl	passive nominal subjects per clause	Clause Complexity
21	parataxis_per_cl	instances of parataxis per clause	Clause Complexity
22	pcomp_per_cl	clausal prepositional complements per clause	Clause Complexity
23	prep_per_cl	prepositions per clause	Clause Complexity
24	prt_per_cl	phrasal verb particle per clause	Clause Complexity
25	tmod_per_cl	bare noun phrase temporal modifiers per clause	Clause Complexity
26	xcomp_per_cl	open clausal complements per clause	Clause Complexity
27	xsubj_per_cl	controlling subjects per clause	Clause Complexity
28	advmod_per_cl	adverbial modifiers per clause	Clause Complexity
29	aux_per_cl	auxilliary verbs per clause	Clause Complexity
30	auxpass_per_cl	passive auxilliary verbs per clause	Clause Complexity
31	modal_per_clause	modal auxilliaries per clause	Clause Complexity
